# CRISPR-Cas9 gene editing and human diseases

**DOI:** 10.6026/973206300181081

**Published:** 2022-11-30

**Authors:** Chaitra Jinka, Chithirala Sainath, Shyamaladevi Babu, Ashok Chakravarthi Chennupati, Lakshmi Prasanna Muppidi, Madhan Krishnan, Gayathri Sekar, Mayilvanan Chinnaiyan, Swapna Kumari Andugula

**Affiliations:** 1 Department of Animal Biotechnology, Sri Venkateswara University, Tirupati - 517502; 2 Syngene International Limited, Biocon park, Bangalore, Karnataka, India; 3 Department of Zoology, Sri Venkateswara University, Tirupati- 517502; 4 Faculty of Allied Health Sciences, Chettinad Hospital and Research Institute, Chettinad Academy of Research and Education, Kelambakkam-603103, Tamil Nadu, India; 5 Department of Physiotherapy, KIMS College of Physiotherapy, KIMS and RF, Amalapuram, Andhra Pradesh; 6 Department ofObstetrics and Gynecological Nursing, KIMS College Of Nursing, KIMS and RF, Amalapuram; 7 Department of pathology, Biosciences research Foundation, Chennai, Tamilnadu, India; 8 Department of Otolaryngology, University of Oklahoma Health Sciences Center, Oklahoma City, USA; 9 KIMS College of Nursing, KIMS and RF, Amalapuram, East Godavari district, Andhra Pradesh, India

**Keywords:** CRISPR-Cas9, ICH, gene editing, viral vector system, on-viral vector systemv

## Abstract

CRISPR/Cas-9 mediated genome editing has recently emerged as a potential and innovative technology in therapeutic development and biomedical research. Several recent studies have been performed to understand gene modification techniques in obtaining effective ex vivo results. Generally, the disease targets for gene correction will be in specific organs, so understanding the complete potential of genomic treatment methods is essential. From such a perspective, the present review revealed the significant importance of the CRISPR/ Cas9 delivery system. Both the promising gene-editing delivery systems, such as synthetic (non-viral) and viral vector systems are discussed in this review. In addition, this paper attempted to summarize the tissue-specific and organ-specific mRNA delivery systems that provide possible research information for future researchers. Further, the major challenges of the CRISPR/Cas9 system, such as off-target delivery, immunogenicity, and limited packaging, were also elucidated. Accordingly, this review illustrated a wide range of clinical applications associated with the efficient delivery of CRISPR/ Cas9 gene-editing. Moreover, this article emphasizes the role of the CRISPR/Cas9 system in treating Intra Cerebral haemorrhage (ICH), thereby suggesting future researchers to adopt more clinical trials on this breakthrough delivery system.

## Background:

The pervasiveness of biomedical research constantly aims at developing an efficient treatment for genetic diseases. CRISPR, which is the natural component of the immune system of bacteria, stands for Clustered Regularly Interspaced Short Palindromic Repeats (CRISPR) [1, 2]. It is considered as the hallmark of the effective bacterial defence mechanism, and the prokaryotes have greatly taken the advantage of this survival tool for self-protection by degrading the invading plasmid DNA or bacteriophage DNA.Concurrently, the discrimination between self and non-self DNA has been effectively performed by screening exogenous DNA from the internal CRISPR loci [3]. These significant characteristics form the basic criteria for CRISPR CAS9 gene-editing technique.In this method, CRISPR is biologically programmed to target the specific genetic code and edit the fault DNA at specific locations. This system could be effectively explored and utilized for other purposes, such as disease diagnosis. With the employment of CRISPR, researchers can perform permanent gene modification in living organisms, thereby correcting mutations at a specific location.Genome editing is a method that performs DNA mutation by inserting or deleting, or base substituting in the targeted gene of interest. Generally, Gene editing consists of several techniques that use transcriptional activator-like effector nuclease, Zinc Finger Nuclease (ZFN) and CRISPR system. Among these, extensively employed system, ZFN, in which the targeted DNA cleavage proteins was used to degrade the DNA sequences at any location. Similarly, Transcription Activator-like Effector Nucleases (TALEN) causes double-stranded breaks in the targeted regions that stimulate DNA damaging pathways and lead to genetic modification. Further, there is low specificity with ZFN, so it is associated with the frequent off-target mutation. Additionally, vector construction of TALEN and ZFN has been more labour-intensive and time consuming [4]. Apart from this specified limitation, the complex genetic patterns of the human genome hinder the efficiency of prevailing tools. Hence, it is necessary to focus on optimizing tools like emerging CRISPR variants [5]. The CRISPR system is efficiently guided by RNA endonucleases, targeting the desired DNA sequences through the nucleotide base pairing. Due to such interesting characteristics, the present review reveals the applications of the CRISPR system in various diseases, especially intracranial haemorrhage. The review also focused on mentioning the target specificity and efficiency of the CRISPR system, followed by an enumerative discussion of the challenges and future prospectus.

## Mechanism of CRISPR Cas 9 system:

The mechanism of the CRISPR/Cas system is categorized as type I to VI, in which type I system utilizes cas3, which is a protein containing both helicase domain and DNase for degrading the targeted DNA. Similarly, the type II system uses Cas9, Cas 2 and Cas 1 associated with the use of Csn2 or Cas4 to break the nucleotides [6]. Subsequently, the type III CRISPR system consists of Cas10, which performs a distinct role. The type II system has been observed to be originated from the organism S. pyrogens and consists of three major components, which are

Cr RNA (CRISPR RNA)

Trace RNA(transactivation RNA)

Cas9 protein

 The Cas9 possesses DNA-cleavage domains like HNH (His/Asn/His) and Ruv C (Recombination UV C) that degrades the ds DNA site situated three base pairs upstream of PAM sequences in the targeted DNA. Here Ruv C domain cleaves the opposite strands of ds DNA and the HNH domain cleaves the complementary crRNA strand. Due to this, the DNA is repaired in-vivo by homology-directed repair termed HDR or non-homologous end joining termed (NHEJ), which are considered error-prone [6]. Here the HDR performs precise gene replacement or accurate insertion of the sequence by adding a homologous donor DNA template at the determined site. Then the DNA is cleaved effectively by establishing ribo nucleoprotein (consisting of tracrRNA and crRNA) by Cas9 [7].

## cr RNA:

It performs a vital role in recognizing and matching the targeted DNA and comprises of a sequence for guiding Cas9 RNP to form R loop at the specific locus. Such R-loop formation activates RuvC and HNH in cleaving the non-targeted and the target DNA strands.

## tracrRNA:

It binds to Cas9 protein and crRNA to form a complex. Further, gRNA is developed as a chimeric molecule containing crRNA and tracrRNA preceded by 18 to 20 nucleotide spacer sequence that is complementary to the target located near to PAM. The PAM plays a vital role in Cas9 mediated DNA degradation. CRISPR system follows the below specified main steps for gene editing

 Expression of Cas9 protein

Generation of gRNA consisting 20 nucleotide (complement to gene of interest)

 Recognition of NGG PAM site situated adjacent to 3ʹ end

With the guidance of sgRNA, Cas9 explores the target in the genome and generates blunt end double stranded base-pairing at 3bp upstream of the PAM region.

## Delivery system:

The delivery strategies of CRISPR components comprising g RNA and Cas nuclease into the target cells is through the following formulations [8].

## DNA:

Here two plasmids are present, in which one encodes a protein and the other encodes a gRNA or a single plasmid that encodes both the components.

## RNA:

Cas protein with mRNA and g RNA as an in-vitro transcribed component.

## Protein:

gRNA-RNP complex and Cas Protein; Synthetic gRNA component and Cas Protein; The following table 1 provides the comparison of features between different delivery modes

## Viral Delivery System:

The viral vectors remain to be promising system in in-vivo CRISPR/Cas9delivery. Because of their huge cellular uptake and editing effectiveness, the viral vectors are extensively used for gene editing. Recently the most widely used viral vectors are lentiviral vectors, adeno associated viruses, adenoviral vectors [9]. These viral vectors deliver CRISPR systems to several tissues or organs through various routes.

## Non-Viral delivery systems:

The progression of synthetic vectors enrolled non-viral delivery systems with a transformative impact on the gene editing field to drive the treatment applications. They possess a flexible packaging capability and hence it could accommodate huge nucleic acids and protein cargos like Cas9 mRNA, sg RNA, donor DNA and RNP [10]. By combining these substances into a nanoparticle (NP), immunogenicity would be reduced and hence nanoparticles should be repeatedly administered. Further use of such NP is transient and hence it decreases the chances of insertional mutagenesis. Hence the fear of nuclease induced off-target events is highly ruled out.

## Intracranial injection:

Apart from viral delivery, there exists a considerable interest in the development of non-viral vectors that could perform gene editing in the intracranial region of the brain.

## ICH and crispr-cas9 system:

Intra-cerebral haemorrhage (ICH) is considered a crucial neurological disease associated with high mortality and morbidity of the entire stroke subtypes. The risk factors associated with ICH comprise hypertension, alcohol use, usage of oral anticoagulants and cigarette smoking. There exist no proven efficient surgical or medical treatments available for ICH. However, cerebellar haemorrhage decompression has been widely accepted as a potential lifesaver. More experimental evidence depicted that the blood-brain barrier (BBB) is one of the key aspects of brain injury and ICH. Raised level of BBB permeability causes vascular-derived oedema after ICH. Treatment methods against BBB disruption attenuate secondary brain damage after ICH. Thus the existing treatment strategies against BBB disruption would help attenuate secondary brain damage after ICH. The role of Frizzled 7 signal to preserve BBB post-ICH in the CD 1 mouse model was investigated by [11]. Activation or knockdown of Frizzled 7 has been done by administering CRISPR through intra-cerebro-ventricular injection before 48 hours of ICH induction. This study performed included WISP activation or knockdown for assessing the signalling pathway. The post–ICH evaluation comprised brain oedema, neuro behaviour, BBB permeability, western blotting, haemoglobin level and immunofluorescence. The research concluded that the FZD7 activation improved BBB disruption, brain oedema and neurological impairments in mice after ICH. Such neuro protection directed by CRISPR has been suggested to obtain advanced treatment in ICH.

The defects in multiple-cell signal pathways cause vascular integrity disruptions that necessitate the optimized regulation of cell adhesive complexes. Such defects were associated with the progression of ICH. Genetic evidence for ICH is observed in humans and also the signaling of adaptor proteins plays a vital process in cell signaling pathways. The study [12] is investigated in a zebra fish for null mutant in iqgap gene by deleting 11bp using CRISPR-Cas9 gene-editing technique. This is followed bycharacterizing its phenotype. The homozygous mutants reveal extreme morphological abnormalities and brain hemorrhages that are lethal in nearly 40% of the cases. The study visualized the expression pattern of iqgap 1 corresponding to the marker and observed a strong overlapping and more expression in muscle, caudal hematopoietic tissue and bronchial arches. Furthermore, the research found that iqgap1 displayed co-localization with fli1a, a marker in the blood vessels of CNS, where the disruption is responsible for hemorrhage. Accordingly, this technique is suggested to detect the ameliorating and intensifying modifier lesion, which in turn aims to develop strong therapeutic agents for restoring normal vascular integrity and thereby preventing ICH.

The presented review also observed that C-C chemokine receptor type 1 (CCR1) and CCL 5, its associated ligand which were responsible for the progression of ICH. These compounds actively participate in the pathogenicity of neuro inflammatory diseases. The CCL/CCR1 signaling in the permeability of blood brain barrier after ICH remains completely unrevealed. Motivated by the above description [13], 250 CD1 male mice were used for ICH induction through injecting autologous whole blood. This experiment administered a selective antagonist of CCR1 integrated with CRISPR activator to the ICH induced mice after a time period of one hour. The research conducted post ICH assessments such as brain water content, neurobehavioral tests, western blotting and hematoma volume. The findings of the study demonstrated that CCR5 inhibition improved by the CRISPR system after inducing ICH and hence the suggested article revealed the therapeutic implication of CRISPR based system in the management of ICH patients. Another risk factor observed in ICH patient is the brain arterivenous malformation, in which its complete pathogenesis has not been revealed. Existing studies detected the somatic mutations in bAVM and accordingly a feasible tool was developed by [14] to generate the sporadic bAVM in the laboratory animals. For the purpose, the authors used CRISPR/Cas9 method for inducing somatic gene mutations in the brain of mouse.

Similarly, integration of CRISPR/CAS 9 with the desired single-cell electroporation method for gene editing in the single neurons in-vivo was performed by [15]. This study suggested using CRISPR Cas9 induced knockout gene in the Purkinje cells of the cerebellum. The study's results demonstrated the gene-editing feasibility of single brain cells that suggested the CRISPR system's proliferative utilisation for neurological disorders' molecular perturbations.

## The existing studies have the following limitations:

 First, the prevailing research has not explored the process of underlying endogenous marker expression after ICH briefly [16].

 After the induction of ICH, all kinds of neuro-protective systems comprising neurons, pericytes, and astrocytes contributing to the injury were not completely elucidated.

 The impacts of the signalling process on the hematoma resolution and angiogenesis after inducing ICH were also not fully investigated.

 The general studies did not provide adequate information about the leakage of substances with smaller molecular weight, and hence the extent of leakage could not be determined successfully.

## Tissue-specific and organ specific mRNA Delivery and CRISPR-Cas gene editing:

Due to lack of carriers for systematic delivering of RNP, several therapeutic targets could not be accessed. They reported generalized methods for engineering the altered lipid nanoparticles to efficiently deliver RNPs to the cells. Following this process, tissue editing comprising brain, muscle, lungs and liver has been accomplished. The intravenous injection enables tissue specific mRNA delivery into the lungs of mouse. A high carrier potential has been leveraged for developing organ specific cancer mo the lungs and livers of mice. The authors [17] also reported that the developed carriers delivers RNPs for restoring dystrophin expression in the DMD mouse model that considerable decreases the serum level. This method can be utilized to develop nanoparticle based drug delivery system based on accurate genetic correction approaches and protein delivery [15]. CRISPR/Cas gene editing and mRNA based delivering system plays a tremendous role to effectively handle disease leading mutations. But it is not impossible to design the vector or nanoparticles for selectively targeting particular tissues. This [16] study reported a Selective organ targeting (SORT) method where sever lipid NP are engineered systematically for exclusively editing the extra hepatic tissues by adding supplemental SORT molecule. To greatly reveal the greater potential of CRISPR Cas9 system, [17]deliberated that iPLNP developed in large scale with the use of microfluid mix possess effective and organ specific delivery. It also allowed repeated injection and observed to be safe at experimental doses. This paper improved the role of phospholipids from unexplored helper components of the lipid nanoparticles to perform a vital role. Thereby significant expansions of the LNP optimization methods are explored. Finally the study suggested that the organ specific CRISPR/ Cas gene editing method possess numerous applications for human genetic therapy was suggested by Yan et al. (2021) [18]. A nano assembly Nano-Pro- Cas9 system the combines the targeted delivery and induced CRISPR Cas9 developed, offers a site specific precise therapeutic gene editing for inflammatory diseases.This system prevents off target mutations at the non- targeted sites which reduce the potential gene toxicity in the organs.The paper suggested that this system could be used to treat many other inflammatory diseases [18].

## Overall challenges:

Despite the effectiveness of CRISPR/Cas 9, this section describes the potential limitations of the investigated system.

There exists an inherent danger, such that it is developed to cleave the targeted genomic site, but it would cleave the other sites and leading to off-target mutations [19]. Generally off target mutations occurs at the regions that looks similar to on target site. Another major difficulty is the prediction of preciseness of occurring off target site [20].

 Despite the occurrence of off target mutations by CRISPR/Cas9 system, it is found to be very rare, even single cell mutations among the potentially targeted billion of cells leads to more feared complications.

 Recently there is no strong evidence to prove the safety of the clinical applications of CRISPR/Cas 9. To provide such information, various unbiased methods for gene scanning for billions to millions of cells were adopted, but too went in vain due to constrained cultured cells or test tubes in vitro [21].

 The researchers determined that the gene editing with Cas 9 protein could be proved effectively at in-vivo conditions,since questions arise regarding the long term safety in human tissues. So optimization in terms of clinical trials seems to be complex [22].

Meanwhile the first CRISPR/Cas9 therapy were performed in sick patients who are supposed to face multiple risks[23].

Although if CRISPR Cas 9 could create a DNA break at a high efficiency site by introducing a particular alteration, efficiency at HDR site is found to be minimum.

 Further for proliferating active HDR cells, it is complex to ensure that cells utilize HDR to repair the DNA breaks [24].

 In addition, since NHEJ is observed to be active in all the cell, large number of cells with NHEJ mediated disruption of target gene were found [25].

This cause high problem during the correction of a gene that causes disease.

## Applications of CRISPR cas9:

There are clinical trials in using CRISPR/cas9 targeting CD19 to remove TRAC and HLA-I to cure Non-Hodgkin lymphoma and acute lymphocytic leukemia [26, 27, 28], which are in the middle of Phase I clinical trials happening in China. The CRISPR/cas9 gene-editing technology is used by targeting CD19 to remove TRAC and B2M, to cure B cell leukemia and lymphoma [28], which are in the middle of phase I clinical trials in China. The same CRISPR/cas9 is used in curing RR and newly Diagnosed high-risk acute myeloid leukemia 28] by targeting CD19 to remove TCR and CD52, to cure which are in the middle of phase I clinical trials in China. Also, using CRISPR/cas9 to cure multiple myeloma by targeting BCMA to remove TRAC and HLA-I is in the middle of phase I clinical trials in China.

CRISPR/Cas9 can reduce the resilience of tumours against these molecular targeted drugs or inhibitors by editing genes related to EGFR, ALK, and KRAS, which would support lung cancer patients. The CRISPR/Cas9 technology evaluates the gene targeted by drugs used in chemotherapy and identifies new pathways to reduce or eliminate resistance to chemotherapy in lung cancer. Also, the CRISPR/Cas9 technology used to edit proto-oncogenes or tumour-suppressor genes provides a great opportunity in lung cancer treatment [29]. CRISPR/Cas9 technology reduces HIV-1 infection by targeting the HIV-1 genome, which clears the provirus. This also involves in the elimination of latent virus, which is activated by provirus removal and has seen successful applications in human cells and animal models [30].

## Conclusion:

Inducible CRISPR Cas System has also to be tailored and understood deeply for adopting extensive application in clinical scenario. This paper suggested that RNA editing and epigenetic regulation is much required apart from prevailing gene editing The present review offered new in-depth insights about the viral and non-viral based CRISPR based systems to promote safe and precise genome editing techniques. Since the number of pathological and physiological signals like pH, redox and ATP could be leveragedfor developing various types of gene editing tools, the presented article will functions as a new gateway to such future researchers. The overall challenges provided in this paper will alarm the researchers thereby helps to avoid pre-existing mistakes.
